# Factors influencing presentation delay among cancer patients: a cross-sectional study in Malaysia

**DOI:** 10.1186/s12889-024-18643-2

**Published:** 2024-05-08

**Authors:** Tshewang Gyeltshen, Hoon Shien Teh, Ching Ee Loo, Nicholas Yee Liang Hing, Wei Yin Lim, Shridevi Subramaniam, Wen Jun Wong, Zoie Shui-Yee Wong, Wen Yea Hwong

**Affiliations:** 1 Graduate School of Public Health, St. Luke’s International University, Tokyo, Japan; 2 Division of Surveillance and Policy Evaluation, National Cancer Center Institute for Cancer Control, Tokyo, 104-0045 Japan; 3 Centre for Clinical Epidemiology, Institute for Clinical Research, National Institutes of Health, Ministry of Health, Shah Alam, Malaysia; 4 Julius Center for Health Sciences and Primary Care, University Medical Center Utrecht, Utrecht University, Utrecht, the Netherlands; 5 Ministry of Health, Royal Government of Bhutan, Thimphu, Bhutan

**Keywords:** Presentation delay, Cancer screening, Malaysia

## Abstract

**Background:**

Cancer represents a significant global public health challenge, with escalating incidence rates straining healthcare systems. Malaysia, like many nations, has witnessed a rise in cancer cases, particularly among the younger population. This study aligns with Malaysia’s National Strategic Plan for Cancer Control Programme 2021–2025, emphasizing primary prevention and early detection to address cancer’s impact. Therefore, we aim to describe the timeliness of cancer care for symptom presentation, socio-demographic, patient, as well as organizational-related factors among patients in Malaysia diagnosed with breast, colorectal, nasopharyngeal, and cervical cancer.

**Methods:**

This cross-sectional study enrolled adult cancer patients diagnosed with breast, cervical, colorectal, or nasopharyngeal cancer from 2015 to 2020 in seven public hospitals/oncology centres across Malaysia. Data were collected through patient-administered surveys and medical records. Presentation delay, defined as the duration between symptom onset and the patient's first visit to a healthcare professional exceeding 30 days, was the primary outcome. Statistical analysis included descriptive statistics and chi-square tests.

**Results:**

The study included 476 cancer patients, with breast cancer (41.6%), colorectal cancer (26.9%), nasopharyngeal cancer (22.1%), and cervical cancer (9.5%). Over half (54.2%) experienced presentation delays with a median interval of 60 days. Higher proportions of presentation delay were observed among nasopharyngeal cancer patients, employed patients with lower socioeconomic statuses, and those without family history of cancer. Most patients self-discovered their first cancer symptoms (80%), while only one-third took immediate action for medical check-ups. Emotional and organizational factors, such as long waiting times during doctor's visits (47%), were potential barriers to seeking cancer care.

**Conclusion:**

This study highlights the significant problem of presentation delay among cancer patients in Malaysia. The delay is influenced by various factors encompassing sociodemographic characteristics, health-seeking behaviours, and healthcare system-related issues. A comprehensive approach addressing both individual barriers and institutional obstacles is imperative to mitigate this presentation delay and improve cancer outcomes.

**Supplementary Information:**

The online version contains supplementary material available at 10.1186/s12889-024-18643-2.

## Introduction

Cancer is recognized as a major public health burden worldwide. Besides being one of the leading causes of mortality and morbidity, increasing trends of cancer over the years has strained health systems across the world. The World Health Organization (WHO) estimates 20 million new cases of cancer and 10 million deaths from cancer-related causes annually. By 2040, the global cancer burden is estimated to grow to 28.4 million new cancer cases and 16.3 million cancer-related deaths [1]. Cancer affects individuals in the prime of their lives, as evidenced by the fact that 57% of newly diagnosed cases occur in individuals aged 69 years or younger [2].

Malaysia reports increasing trends of cancer incidence over the years. Amongst Malaysian women, breast, colorectal and cervical cancers were the top 3 most common cancers for the last 10 years whereas colorectal, lung, prostate and nasopharyngeal cancers topped the list for types of cancers among Malaysian men [3]. This is comparable to the global picture, except for nasopharyngeal cancer which has been observed to have a higher prevalence and incidence in Asia. The International Agency for Research on Cancer in 2020 has shown that more than 85% of patients with nasopharyngeal cancer were from Asia and of that, the highest incidence and mortality for this cancer was among Malaysia men at 7.2 per 100,000 population in 2012.To further complicate our situation, 64% of patients in overall cancers had advanced stages (stage III and IV) at the time of diagnosis and this leads to poor survival rates [4].

To address this issue, it is crucial to advocate primary prevention and early detection of cancers, in line with the focus area of Malaysia’s National Strategic Plan for Cancer Control Programme 2021–2025. Delays in presentation, diagnosis and treatments are well-known to affect cancer outcomes significantly. Advocating for early diagnosis programs, WHO highlights that delays could occur anytime during the cancer trajectory from access to care, evaluation of disease, to access to subsequent treatment [5]. Each of these steps corresponds to an interval: patient, diagnosis, and treatment, respectively. A similar work on breast cancer in Malaysia highlighted that the presentation interval from onset of symptoms to diagnosis is the longest [6].

Besides being setting specific, multiple reasons could have contributed to this delay. Apart from sociodemographic and health system-related factors, reasons contributing to this delay could potentially be explained by a multidimensional chain of behaviours during the process of informed decision-making for seeking medical help after the detection of the first potential cancer symptoms. Several studies have been conducted among breast cancer patients in a qualitative manner locally. A meta-synthesis showed that knowledge, psychological, sociocultural, and health system- related factors influenced the health-seeking behaviour among breast cancer patients in Malaysia. Similar barriers were also highlighted in a study spanning across Malaysia and Singapore [7]. We intend to add further to the current scientific evidence by describing the timeliness of cancer care in four types of cancer namely breast, colorectal, nasopharyngeal, and cervical cancer in Malaysia. The World Health Organization (WHO) recommends the importance of prioritizing strategies for cancers amenable to early diagnosis. Successful treatment is more achievable for solid tumours that are amenable to early diagnosis. Solid tumours such as breast, colorectal, nasopharyngeal, and cervical cancer constitute 40% of all cancer cases in Malaysia and offer better treatment prospects with early detection.

Therefore, in this study, we aim to describe the timeliness of cancer care for symptom presentation, socio-demographic, patient, as well as organizational-related factors among patients in Malaysia diagnosed with breast, colorectal, nasopharyngeal, and cervical cancer.

## Methodology

### Study design and population

Data extracted for this study is part of a larger multi-centre, cross-sectional study which recruited adult cancer patients 18 years and above who were diagnosed with primary cancers of breast, cervical, colorectal, or nasopharyngeal from the year 2015 to 2020. Patients with recurrent or synchronous cancers or with conditions which contribute to cognitive impairment were excluded from the study. This study was conducted across seven public hospitals/oncology centres throughout Malaysia. In brief, Malaysia has a two-tiered healthcare system of public and private healthcare. Adopting the approach of universal healthcare, the public healthcare system is heavily subsidised by the government whereas the co-existing private sector comprises private clinics and hospitals, several Non-Governmental Organizations (NGOs) and other privately-owned health-related services, are funded by either fee-for-service or healthcare insurances. The study was conducted at seven public hospitals in Malaysia. The selection of public hospitals followed a regional approach, with a higher proportion of sites located within the central region. This decision was made after discussions with policymakers and clinicians, considering that these facilities have a higher incidence of cancer cases, with some serving as tertiary referral oncology centres.

### Patient recruitment and data collection

From January 2020 to March 2020, the study team identified eligible patients following the inclusion criteria, using either a clinic outpatient list, completed cancer registry forms or medical records. The sample encompasses all patients deemed eligible during the screening process at study sites, recruited via a convenient sampling based on study criteria. Research assistants approached these potential patients during their clinic appointment dates at study sites, and informed consent has been obtained from all the participants. Eligible patients administered the first section of the survey physically, facilitated by the research assistant. Extraction of medical records for the second section was conducted by the research assistant. Both first and second section data were then entered to the Electronic Data Capture (EDC) system called the Research Electronic Data Capture (REDCap) application to ease data management and analysis in the subsequent steps [8]. A copy of the data collection form was attached to the Supplementary file 1.

### Survey tool

The survey has been developed by referencing a predefined list of variables and subsequently adjusted for cancer-specific related to the four types of cancer. Questions from existing and validated questionnaires were adapted to suit the Malaysian context. The process of item generation and evaluation involved discussions between study team and senior researchers specializing in cancer research to ensure content validity. The survey also underwent face validation with a group of 10 participants to ensure that the questionnaire was appropriately designed and could be self-administered. We also did a pilot test of the survey with 76 people to ensure that the survey was suitable for on-site administration. In this exploratory study, our focus is gaining insights aligned with our study objectives. Hence, reliability testing is considered less critical.

The survey encompasses sections primarily covering socio-demographics and details of the cancer journey, including dates and information on first symptoms, initial presentation, diagnosis and treatment, and social network support. The data collection sheet includes medical records related to diagnosis, staging and treatment. There are a total of 28 questions, with some having sub-questions. The answer options do not have numerical scoring. Respondents were provided with the option to select relevant boxes and fill in dates for specific questions.

### Key variables

The first section of the patient-administered survey included sociodemographic characteristics, source of first cancer symptom recognition of either the detection was picked by patient, family members/friends, healthcare professionals or during health promotional activities, attitude towards cancer symptoms, barriers to consultation such as patient-related emotional and organisation factors, diagnosis, and treatment-related questions. Section two encompassed the details of cancer diagnosis and treatment.

### Definition of interval and delay

An acceptable duration for delay in presentation varies between studies, although it is generally acceptable to be between 1 to 3 months. A delay in presentation in our study is defined as an interval of more than 30 days, based on consensus from literature as well as among local clinicians, which was referred to as presentation delay in this paper. A recent systematic review reported that the median presentation interval did not exceed one month for the majority of the cancer sites among the high-income countries [9]. With a general agreement from the local clinicians on this cut-off point, we decided to benchmark ourselves against what should be the ideally acceptable standard of care globally instead of applying the median patient interval for lower-income countries which were reported to be 1.5 to 4.0 times longer. The small number of studies available for meta-analysis among the lower-income countries was also another factor of why we decided against the choice of at least 1.5 months longer than the high-income countries. If respondents were unable to provide dates for the first symptom or the first visit to the healthcare professional, they were asked to provide a month range. The estimated date would be the midpoint, which was 15th of the month. The estimated dates were verified with the medical notes extraction in "Methodology" section which asked about the duration of symptoms experienced by the patient.

### Statistical analysis

Categorical variables were summarized by frequencies and percentages, and numerical variables were presented as mean or median, interquartile range and standard deviation. We divided the presentation delay into a binary outcome, i.e., delay and non-delay, by using the 30-days cut-off point. The Chi-Square Test of Independence was used to determine the association between the categorical variables. For missing information, the study team contacted the local medical record department staff to extract the necessary information from physical files. Non-available data were deemed missing and were not imputed. A complete case analysis approach was taken for patients with missing presentation intervals and this provides the total number of patients for this study. All the analyses were done using the open software R version 4.3.1.

### Ethics approval

The study has been approved by the Medical Research and Ethics Committee (MREC), adhering to ethical principles outlined in the Declaration of Helsinki and the Malaysian Good Clinical Practice Guideline (NMRR-19–727-47742).

## Results

This study included a total of 476 cancer patients. The median presentation delay was found to be 60 days with an interquartile range [IQR] of 14 – 180 days (Table 1). More than half (54.2%) of patients had a presentation delay of 30 days or more. The mean age at diagnosis was 55.8 ± 11.9 years and it was similar between both groups of presentation intervals. There were 198 patients (41.6%) with breast cancer, 128 patients (26.9%) with colorectal cancer, 105 patients (22.1%) with nasopharyngeal cancer and 45 patients (9.5%) with cervical cancer. A larger proportion of presentation delay was shown among patients with nasopharyngeal cancers (27.5% delay versus 15.6% non-delay) and cervical cancers (9.7% delay versus 9.2% non-delay). Among the patients, the majority were Malay (48.7%), followed by Chinese (40.8%), 8.6% were Indian, and the remaining 2% belonged to other ethnicities. Regarding socioeconomic distribution, most of the patients were from the middle-class with secondary school qualifications. Presentation delay was slightly higher in the employed group with income below RM6000. About 60% of the patients did not have a family history of cancer. Nearly two thirds of the patients with presentation delay were diagnosed with late-stage cancer compared to about 50% of patients with late-stage cancer among those without presentation delay. It is noteworthy to point out that those who did not have a family history of cancer and were diagnosed with late stage of cancers had a higher tendency to present their symptoms late.

**Table 1:** Sociodemographic characteristics of the study participants

**Sociodemographic characteristics**	Presentation Delay ≤ 30 days	Presentation Delay > 30 days	**Total, *N* = 476**
	**n (%)**	**n (%)**	**n (%)**
Presentation interval, median (IQR)	-	-	60 (166)
	218 (45.8)	258 (54.2)	
**Age at Diagnosis, years**			
Mean (SD)	56.8 (11.5)	55.0 (12.2)	55.8 (11.9)
Range	24.4—85.5	19.2—82.7	19.2—85.5
**Cancer Type**			
Breast	100 (45.9)	98 (38.0)	198 (41.6)
Colorectal	64 (29.4)	64 (24.8)	128 (26.9)
Nasopharyngeal	34 (15.6)	71 (27.5)	105 (22.1)
Cervical	20 (9.2)	25 (9.7)	45 (9.5)
**Ethnicity**			
Malay	110 (50.5)	122 (47.3)	232 (48.7)
Chinese	78 (35.8)	116 (45.0)	194 (40.8)
Indian	25 (11.5)	16 (6.2)	41 (8.6)
Others	5 (2.3)	4 (1.6)	9 (1.9)
**Education Level**^a^			
No Formal Education	6 (3.6)	14 (7.1)	20 (5.5)
Secondary school	163 (74.8)	193 (74.8)	356 (74.8)
Tertiary Education	49 (29.5)	49 (24.9)	98 (27.0)
**Employment status**			
Employed	118 (54.1)	154 (59.7)	272 (57.1)
Unemployed	63 (28.9)	68 (26.3)	131 (27.6)
Retiree	37 (17.0)	36 (14.0)	73 (15.3)
**Household Income**^a^			
RM0 – RM999	42 (19.3)	42 (16.3)	84 (17.6)
RM1000 – RM3000	103 (47.2)	126 (48.8)	229 (48.1)
RM3001 – RM6000	41 (18.8)	69 (26.7)	110 (23.1)
RM6001 and above	31 (14.2)	16 (6.2)	47 (9.9)
**Family history of cancer**			
Yes	91 (41.7)	91 (35.3)	182 (38.2)
No	127 (58.3)	167 (64.7)	294 (61.8)
**Cancer staging**^a^			
Stage 1	26 (13.3)	20 (8.2)	46 (10.5)
Stage 2	71 (36.2)	63 (25.8)	134 (30.5)
Stage 3	64 (32.7)	103 (42.2)	167 (38.0)
Stage 4	35 (17.9)	58 (23.8)	93 (21.1)
**Presence of comorbidity**			
Yes	84 (38.5)	104 (40.3)	188 (39.5)
No	134 (61.5)	154 (59.7)	288 (60.5)

^a^The differences are due to missing values

Table 2 shows that more than 80% of the patients self-discovered their first cancer symptom. Another 11% of patients had their symptoms detected by either the healthcare professionals or via health screening whereas less than 6% had their family members or relatives finding out their first cancer symptom. Patients with symptoms detected by healthcare professionals had a lower percentage of presentation delays, compared to the other means of symptom recognition. Furthermore, only one-third of the patients had an instant early intentional action for proper medical check upon discovering symptoms. Majority of the patients chose to deny the symptoms (36.8%) or ignore the problems (28.6%), and these have contributed to a larger proportion of presentation delay.

**Table 2:** Symptoms recognition and attitudinal factors associated with presentation delay

**Symptoms Recognition and Attitudinal Factors**	Presentation Delay ≤ 30 days, n (%)	Presentation Delay > 30 days, n (%)	**Total (476) N (%)**
***Symptoms Recognition***			
*Symptoms detected by doctor/health promotional activities*	34 (15.6)	17 (6.6)	51 (10.7)
*Symptoms detected by me*	175 (80.3)	222 (86.0)	397 (83.4)
*Symptoms detected by family members/friends*	9 (4.1)	19 (7.4)	28 (5.9)
***Attitudinal Factors***			
*I may have cancer. I need to get myself checked*	81 (37.2)	73 (28.3)	154 (32.4)
*It is not cancer. The symptoms appeared because of something else*	78 (35.8)	97 (37.6)	175 (36.8)
*I thought it was not serious. The problem or symptom will go away soon*	51 (23.4)	85 (32.9)	136 (28.6)
*Other thoughts*	8 (3.7)	3 (1.2)	11 (2.3)

**Fig. 1:**
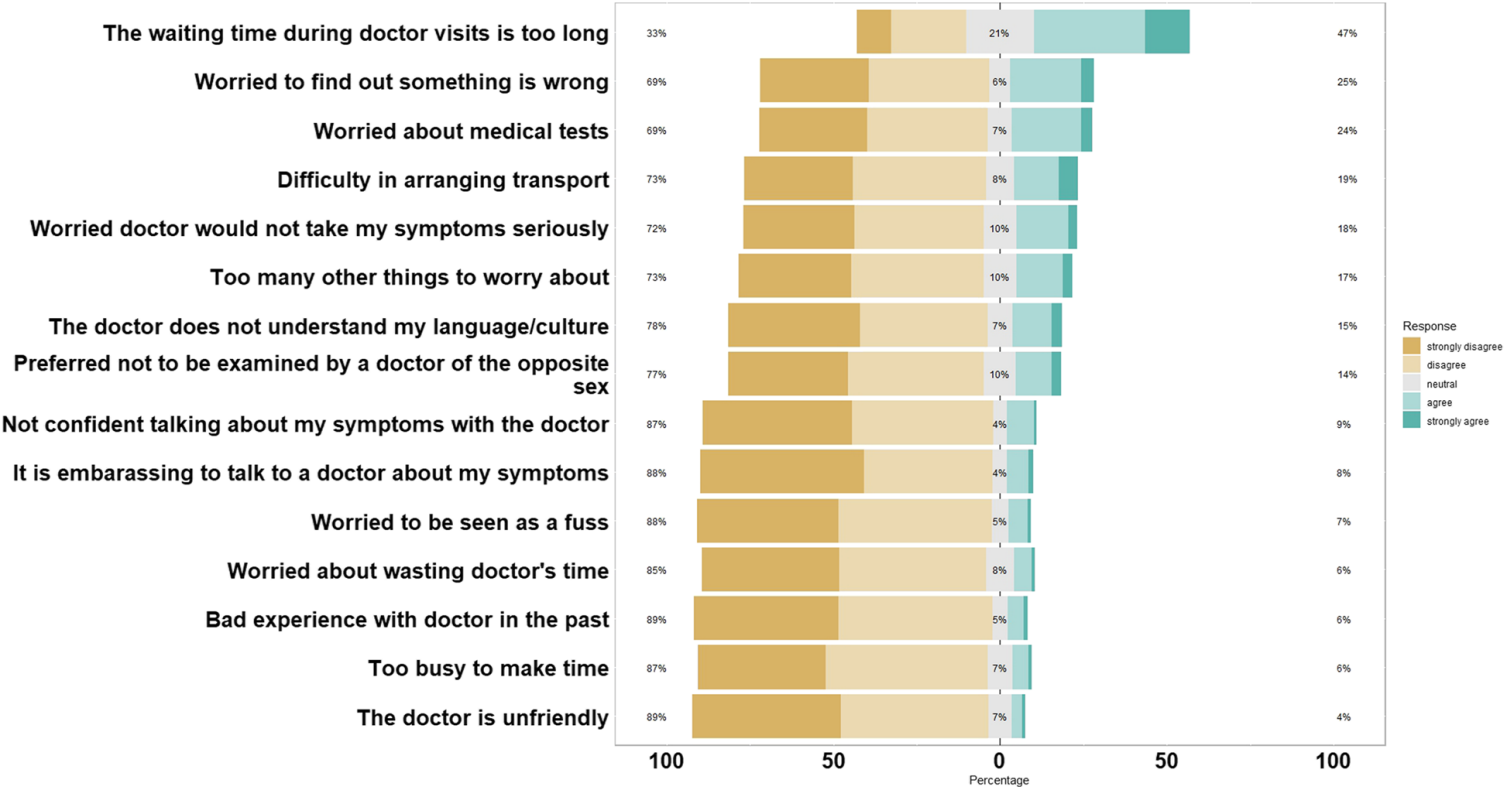
Emotional and organisational factors of the study participants (*N* = 476)

The emotional and organisational factors contributing to presentation delay were displayed in Fig. 1. Although none of these factors reached statistical significance, the top three perceived barriers to seeking cancer care included concerns about prolonged waiting times during doctor's appointments (47%), worrying about receiving unfavourable results (25%), and anxiety regarding follow up medical tests (24%).

## Discussion

More than half of the cancer patients (54.2%) experienced a delay in their presentation interval. The median time from symptom recognition to seeking consultation was 60 days, with an interquartile range (IQR) spanning from 14 to 180 days. In parallel to our results, the consecutive Malaysian National Cancer Registry Report of 2007—2011 and 2012 – 2016 showed that the cancers diagnosed are mostly at the stages of III and IV, indicating significant delay in presentation intervals [10]. Higher proportions of presentation delay were also observed among nasopharyngeal cancer patients, employed patients with lower socioeconomic statuses, and those without family history of cancer. Importantly, although over 80% of the patients independently noticed their initial cancer symptoms, only one-third of them promptly decided to seek medical attention. Several key emotional and organisational factors associated with this delay included extended wait times for doctor’s appointments, apprehension about receiving unfavourable results, and concerns regarding the nature of medical tests doctors might perform next.

Presentation delay is a long-known challenge in cancer care globally. There is an undeniable influence of a country’s socioeconomic status on the timely access to cancer care. Petrova et al. reported a pooled median presentation interval of 30 days (a month) for most cancers in high-income countries whereas in lower-income countries, the interval is 1.5 to 4 times longer. This divergence in presentation intervals was further affirmed by another systematic review, highlighting patient presentation intervals to be significantly longer in low-income countries with a median of 6.5 months [11]. Comparing our intervals with others, a study on cervical cancer conducted in Nepal revealed similar findings to ours with a median of 68.5 days between symptom recognition and initial presentation. Another local study on colorectal cancer in 2009 found that patients waited 13 weeks before seeking medical consultation. While this may suggest that colorectal cancer awareness and education in Malaysia may have improved over the years, it is important to acknowledge that the challenge of presentation delay in our country remains huge with over 50% of our patients presenting to the healthcare after 30 days. Crucially, this has direct implications on the cancer stages and consequently, the patient's prognosis and survival [12].

We showed that nasopharyngeal cancer patients had a higher tendency for presentation delay. Concurring with the findings from the local national cancer registry that about 65–69% of nasopharyngeal cancer patients were in Stage III and IV upon diagnosis, nasopharyngeal cancer is well known for its atypical and non-specific initial symptoms [13]. Variations in its symptoms also potentially contribute to the difficulty of recognising the need to seek healthcare.

Our study indicated that most cancers were diagnosed following patients’ self-report of cancer symptoms. This underscores the individual’s ability to self-recognize when their symptoms are beyond their control and necessitate professional help. While our analysis did not directly measure cancer-related knowledge, it is noteworthy that a substantial percentage of patients reacted to their symptoms with immediate negative attitudes, which could stem from either ignorance or denial of the potential seriousness of the symptoms. This suggests a potentially low perceived likelihood of having cancer, which was potentially amplified with the fact that they did not have any family history of cancer. This observation aligns with prior local research, where patients frequently attributed their symptoms to benign ailments or other health conditions [14].

Cancer services in Malaysia predominantly rely on public healthcare services, where long waiting time has been an ongoing concern due to substantial workload and limited human resources [15]. As observed from our findings, this issue becomes particularly relevant when comparing socioeconomic groups. Unemployed individuals may experience fewer obstacles when waiting for consultations compared to the other employed counterparts with work commitments, which can explain their lower percentage of experiencing presentation delays. Conversely, individuals with lower socioeconomic status and associated work commitments might face greater challenges in accessing consultations promptly.

Besides that, patients’ concerns about discovering a potential health issue and apprehension regarding invasive medical tests were identified amongst the important emotional barriers to timely presentation. These findings underscore the urgent need for proactive public awareness campaigns and community advocacy efforts, as demonstrated by earlier publications [16]. Additionally, addressing the lack of accessible pathways for cancer-related information is crucial for promoting early diagnosis and treatment. Collaborative efforts involving the upgrade of existing oncologic facilities in partnership with local support groups should be a priority across the country to facilitate better access to essential resources and information.

### Implications for future studies

This study accentuates several key areas that warrant attention when designing health-promoting interventions in the future. Despite the availability of government-subsidized screening services, our findings indicate low uptake, with most patients relying on self-recognition of their symptoms. Addressing this issue requires intensified efforts to expand the reach of cancer screening and tackle the access barriers contributing to presentation delays.

One noteworthy takeaway from our investigation is the prominent role played by patients' reluctance to seek medical evaluation due to fear. Furthermore, it appears that some patients may not perceive their symptoms as serious enough to warrant disclosure to a healthcare professional. While ongoing cancer awareness campaigns exist, there is a pressing need to evaluate the effectiveness of the current programs, particularly in terms of knowledge dissemination among the population, especially those with lower socioeconomic status.

To facilitate this, the implementation of worksite screening and onsite screening through public health offices may be a valuable strategy to enhance accessibility and awareness regarding cancer screening, ultimately promoting early detection and timely intervention.

### Strengths and limitations

The strength of our study lies in the robust methodology employed for patient recruitment, which focused on selecting participants from the primary oncology centres within the public healthcare system. While patients from private healthcare facilities were not included, it is important to note that public healthcare dominates more than 90% of outpatient attendances in Malaysia, making our study particularly relevant for informing future interventions [15]. Additionally, we achieved a high completion rate for our survey by utilizing a face-to-face patient-administered approach, enhancing the reliability of our data collection.

Nevertheless, there are certain limitations to consider. One notable limitation is the lack of consistent definitions for presentation delay in the literature, leading to substantial heterogeneity in measured intervals across different publications. Despite this challenge, we made efforts to define the interval using both evidence from prior literature in line with consensus among physicians who lead local medical practices. Convenience sampling was employed in our study due to practical considerations, however respondents from diverse sociodemographic backgrounds were included to enhance the generalizability of our findings. Potential recall bias might be present, which was handled by checking the medical notes where needed and available.

## Conclusion

Our study sheds light on a concerning issue: over half of our cancer patients face a significant delay between recognizing their symptoms and seeking healthcare (presentation delay). This delay is likely to stem from a complex interplay of factors, encompassing patient-related sociodemographic variables, individual’s health-seeking behaviours concerning cancer screening, and systemic organizational challenges within the healthcare system. Addressing these challenges requires a concerted effort through holistic approaches that tackle both personal barriers and institutional obstacles.

### Supplementary Information

**Supplementary Material 1. **

## Data Availability

Data and materials available from the corresponding author upon reasonable request.
