# Supplementary material for: Factors influencing presentation delay among cancer patients: a cross-sectional study in Malaysia
Source: BMC Public Health. 2024 May 8;24:1260. doi: 10.1186/s12889-024-18643-2 (PMC11077827; doi:10.1186/s12889-024-18643-2)
Supplement: Supplementary file 1 — Supplementary Material 1. [file 12889_2024_18643_MOESM1_ESM.pdf]

SECTION 1

SUBSECTION 1: ABOUT YOU

Q1. What is your ethnicity?

- ☐ 1 Malay
- ☐ 2 Chinese
- ☐ 3 Indian
- ☐ 4 Orang Asli Peninsular Malaysia
- ☐ 5 Serani
- ☐ 6 Iban
- ☐ 7 Kadazan
- ☐ 8 Dusun
- ☐ 9 Bidayuh
- ☐ 10 Melanau
- ☐ 19 Other Bumiputera Of Sabah, please specify: ________________________
- ☐ 29 Other Bumiputera Of Sarawak, please specify: ________________________
- ☐ 99 Others, please specify: ________________________

Q2. What is your marital status?

- ☐ 1 Single
- ☐ 2 Married
- ☐ 3 Separated
- ☐ 4 Widow / Widower
- ☐ 5 Divorced
- ☐ 6 Cohabiting
- ☐ 99 Others, please specify: ___________________________

Q3. Do you have any children?

- ☐ 0 No
- ☐ 1 Yes, **how many children do you have?** _______

  How old is your youngest child? ______

  How old is your oldest child? ______

Q4. Who do you live with in your current residence?

- ☐ 1 Living Alone
- ☐ 2 Partner/Family
- ☐ 3 Nursing Home
- ☐ 99 Others, please specify: ___________________________

Q5. What is your highest education level?

- ☐ 0 No formal education / Never been to school
- ☐ 1 Did not complete primary school
- ☐ 2 Completed Standard 6
- ☐ 3 Completed Form 3
- ☐ 4 Completed Form 5
- ☐ 5 Completed Form 6 / Certificate / Diploma
- ☐ 6 Completed Bachelor's Degree
- ☐ 7 Completed Masters
- ☐ 8 Completed Doctoral Qualification (PhD)
- ☐ 99 Others, please specify: ____________________________

SUBSECTION 1: ABOUT YOU (CONTINUED)

Q6. What was your job before being diagnosed with cancer?

☐ 1 Employed, please specify your job: ____

☐ 2 Retiree

☐ 3 Student

☐ 0 Unemployed

Q7. Prior to diagnosis of cancer, how much was your gross monthly personal income (salary / pension)?

☐ 1 RM0 – RM999 ☐ 2 RM1000 – RM3000 ☐ 3 RM3001 – RM6000 ☐ 4 RM6001 and above

Q8. Prior to diagnosis of cancer, what was your monthly household income?

☐ 1 RM0 – RM999 ☐ 2 RM1000 – RM3000 ☐ 3 RM3001 – RM6000 ☐ 4 RM6001 and above

Q9. Have you ever been diagnosed with the following diseases?

Please TICK (✓) all the relevant boxes.

☐ 1 High Blood Pressure

☐ 2 Diabetes

☐ 3 Stroke Related Diseases

☐ 4 Breathing or Lung Related Diseases

☐ 5 Heart Related Diseases

☐ 6 Arthritis / Muscle or Bone Diseases

☐ 7 Longstanding Physical Disability

☐ 8 Cancer, please specify: ___________________________

☐ 99 Others, please specify: ____

Q10. Does any of your family members have history of cancer?

☐ 0 No

☐ 1 Yes, **how are they related to you?** ____

What type of cancer do they have? ____

Q11. Where would you go when you are sick?

Please TICK (✓) all the relevant boxes.

☐ 1 Private Clinic

☐ 2 Private Hospital

☐ 3 Government Health Clinic

☐ 4 Government Hospital

☐ 5 University Clinic

☐ 6 University Hospital

☐ 7 Traditional and Complementary Medicine

**SUBSECTION 2: CONSULTATIONS**					
**Q12. Which of the following <u>might put you off</u> from going to any doctors treating your cancer?** *Please TICK (✓) the most relevant options provided for following statements.*	**Strongly Disagree1**	**Disagree2**	**Neutral3**	**Agree4**	**Strongly Agree5**
**a.** It is embarrassing to talk to a doctor about my symptoms.	☐	☐	☐	☐	☐
**b.** Not confident talking about my symptom(s) with the doctor.	☐	☐	☐	☐	☐
**c.** The waiting time during doctor visits is too long.	☐	☐	☐	☐	☐
**d.** Worried about wasting the doctor's time.	☐	☐	☐	☐	☐
**e.** Worried to be seen as somebody who makes a fuss.	☐	☐	☐	☐	☐
**f.** Worried if the doctor finds out something wrong with me.	☐	☐	☐	☐	☐
**g.** Worried about what medical tests the doctor might want to do.	☐	☐	☐	☐	☐
**h.** Worried the doctor would not take my symptom(s) seriously.	☐	☐	☐	☐	☐
**i.** The doctor does not understand my language or culture.	☐	☐	☐	☐	☐
**j.** Bad experience with doctors' in the past.	☐	☐	☐	☐	☐
**k.** The doctor is unfriendly.	☐	☐	☐	☐	☐
**l.** Prefer not to be examined by a doctor of the opposite sex.	☐	☐	☐	☐	☐
**m.** Difficulty in arranging any mode of transport to the hospital or clinic.	☐	☐	☐	☐	☐
**n.** Too busy to make time to go to the doctor.	☐	☐	☐	☐	☐
**o.** Too many other things to worry about.	☐	☐	☐	☐	☐

SUBSECTION 3: BREAST SYMPTOMS
**Q13.** *Pick ONE (1) option only.* **How did you FIRST notice your cancer symptoms?**
☐ 1 Symptoms detected / picked up by doctor. (**OPTION A**)
☐ 2 Symptoms detected through health promotional activities / health screening. **(OPTION B)**
☐ 3 Symptoms detected by me. **(OPTION C**)
☐ 4 Symptoms detected by family members / friends. **(OPTION C**)

SUBSECTION 3: BREAST SYMPTOMS (CONTINUED)

OPTION A

"Symptoms detected / picked up by the doctor."

Q13.1. What were the reason(s) you saw that doctor for?

____

Q13.2. When did you see this doctor?

___ ___ / ___ ___ / ___ ___ ___ ___ *(DD/MM/YYYY)*

If you cannot remember the exact date(s), please fill in the month and year

Q13.3. Where did you visit this doctor?

- ☐ 1 Private Clinic
- ☐ 2 Private Hospital
- ☐ 3 Government Health Clinic
- ☐ 4 Government Hospital
- ☐ 5 University Clinic
- ☐ 6 University Hospital
- ☐ 7 Traditional and Complementary Medicine
- ☐ 8 Health Screening Centre (e.g. BP Healthcare, Gribbles)
- ☐ 99 Others, please specify: ____________________________

Q13.4. Did this doctor refer you to a specialist / hospital for further investigation of your cancer symptom(s)?

- ☐ 0 No
- ☐ 1 Yes

Q13.5a. Which of the following symptom(s) were detected by this doctor?
Please TICK (✓) all the relevant boxes.

- ☐ 1 Breast lump
- ☐ 2 Nipple problems
- ☐ 3 Breast pain
- ☐ 4 Changes of breast shape
- ☐ 5 Swollen lumps in the armpit
- ☐ 6 Unintentional weight loss
- ☐ 7 Loss of appetite
- ☐ 99 Others, please specify: ___________________________

Q13.5b. When you were told about the above symptom(s), what were your thoughts?

- ☐ 1 I may have cancer. I need to get myself checked.
- ☐ 2 It is not cancer. The symptom appeared because of something else.
- ☐ 3 I thought it was not serious. The problem or symptom will go away soon.
- ☐ 99 Others, please specify: _______________________________________

SUBSECTION 3: BREAST SYMPTOMS (CONTINUED)

Q13.6. Prior to visiting the above doctor, did you visit any other doctor for the above-mentioned symptom(s) (Q13.5a)?

☐ 0 No

☐ 1 Yes, **when was it?** ____ ____ / ____ ____ / ____ ____ ____ ____ *(DD/MM/YYYY)*

If you cannot remember the exact date(s), please fill in the month and year

Q13.7. Prior to visiting the above doctor, have you ever experienced any of the above-mentioned symptom(s) (Q13.5a)?

☐ 0 No

☐ 1 Yes, **when was the first symptom experienced?** ____ ____ / ____ ____ / ____ ____ ____ ____

If you cannot remember the exact date(s), please fill in the month and year. (DD/MM/YYYY)

SUBSECTION 3: BREAST SYMPTOMS (CONTINUED)

OPTION B

"Symptoms detected through health promotional activities / health screening."

Q13.1. When was this health promotional activity?

___ ___ / ___ ___ / ___ ___ ___ ___ *(DD/MM/YYYY)*

If you cannot remember the exact date(s), please fill in the month and year

Q13.2. Where did you visit this health promotional activity?

- ☐ 1 Private Clinic
- ☐ 2 Private Hospital
- ☐ 3 Government Health Clinic
- ☐ 4 Government Hospital
- ☐ 5 University Clinic
- ☐ 6 University Hospital
- ☐ 7 Traditional and Complementary Medicine
- ☐ 8 Health Screening Centre (e.g. BP Healthcare, Gribbles)
- ☐ 99 Others, please specify: ______________________________

Q13.3. Did this doctor <u>refer</u> you to a specialist / hospital for <u>further investigation of your cancer symptom(s)</u>?

- ☐ 0 No
- ☐ 1 Yes

Q13.4a. Which of the following symptom(s) were detected during this health promotional activity?
Please TICK (✓) all the relevant boxes.

- ☐ 1 Breast lump
- ☐ 2 Nipple problems
- ☐ 3 Breast pain
- ☐ 4 Changes of breast shape
- ☐ 5 Swollen lumps in the armpit
- ☐ 6 Unintentional weight loss
- ☐ 7 Loss of appetite
- ☐ 99 Others, please specify: ____________________________

Q13.4b. When you were told about the above symptom(s), what were your thoughts?

- ☐ 1 I may have cancer. I need to get myself checked.
- ☐ 2 It is not cancer. The symptom appeared because of something else.
- ☐ 3 I thought it was not serious. The problem or symptom will go away soon.
- ☐ 99 Others, please specify: ____

Q13.5. Prior to attending the screening program above, have you ever experienced any of the above-mentioned symptom(s) (Q13.4a)?

- ☐ 0 No
- ☐ 1 Yes, **when was the first symptom experienced?** ___ ___ / ___ ___ / ___ ___ ___ ___

If you cannot remember the exact date(s), please fill in the month and year. (DD/MM/YYYY)

SUBSECTION 3: BREAST SYMPTOMS (CONTINUED)

OPTION C

"Symptoms detected by me, family member or friends."

Q13.1. When did you see a doctor?

___ ___ / ___ ___ / ___ ___ ___ ___ *(DD/MM/YYYY)*

If you cannot remember the exact date(s), please fill in the month and year

Q13.2. Where did you visit this doctor?

☐ 1 Private Clinic
☐ 2 Private Hospital
☐ 3 Government Health Clinic
☐ 4 Government Hospital
☐ 5 University Clinic
☐ 6 University Hospital
☐ 7 Traditional and Complementary Medicine
☐ 8 Health Screening Centre (e.g. BP Healthcare, Gribbles)
☐ 99 Others, please specify: ______________________________

Q13.3. Did this doctor <u>refer</u> you to a specialist / hospital for <u>further investigation of your cancer symptom(s)</u>?

☐ 0 No
☐ 1 Yes

**Q13.4a. Which of the following symptom(s) did you experience?** *Please TICK (✓) the relevant boxes and provide the date that you noticed it.*		**DATE** *(DD/MM/YYYY)* *If you cannot remember the exact date(s), please fill in the month and year.*
Breast lump	☐	
Nipple problems	☐	
Breast pain	☐	
Changes of breast shape	☐	
Swollen lumps in the armpit	☐	
Unintentional weight loss	☐	
Loss of appetite	☐	
Others, please specify: ____		

SUBSECTION 3: BREAST SYMPTOMS (CONTINUED)

Q13.4b. When you experienced the above symptom(s), what were your thoughts?

☐ 1 I may have cancer. I need to get myself checked.

☐ 2 It is not cancer. The symptom appeared because of something else.

☐ 3 I thought it was not serious. The problem or symptom will go away soon.

☐ 99 Others, please specify: ______________________________________

SUBSECTION 3: BREAST SYMPTOMS (CONTINUED)

Q14. Who was the first person you talked to about your cancer symptom(s)?

☐ 1 I kept to myself

☐ 2 Partner / Husband / Wife

☐ 3 Other family members

☐ 4 Friends

☐ 99 Others, please specify: _______________________

Q15. What did the person offer you?

Please TICK (✓) all the relevant boxes.

☐ 0 I kept to myself.

☐ 1 Reassurance with words of advice and comfort to ease the worry.

☐ 2 Recommended some alternative medicine (e.g. Supplements).

☐ 3 Suggested for proactive measure such as further medical check-up.

☐ 99 Others, please specify: ____________________________

Q16. Do you have other matter(s) withholding you from getting subsequent cancer check-up / treatment?

Please TICK (✓) all the relevant boxes.

☐ 0 NONE

☐ 1 Take care of young children

☐ 2 Take care of the elderly

☐ 3 Work related

☐ 4 Disability due to existing illness

☐ 99 Others, please specify: ___________________________

Q17. Did you see any other doctors for additional opinion for your cancer symptom(s)?

☐ 0 No

☐ 1 Yes, **how many other doctors?** ____

SUBSECTION 4: CANCER JOURNEY (B: DIAGNOSIS)

Q18. When was your cancer diagnosis first confirmed by a doctor?

If you cannot remember the exact date(s), please fill in the month and year

☐☐ / ☐☐ / ☐☐☐☐

(DD/MM/YYYY)

Q19. Where did you visit this doctor?

☐ 1 Private Clinic

☐ 2 Private Hospital

☐ 3 Government Health Clinic

☐ 4 Government Hospital

☐ 5 University Clinic

☐ 6 University Hospital

☐ 7 Health Screening Centre (e.g. BP Healthcare, Gribbles)

☐ 8 Traditional and Complementary Medicine

☐ 99 Others, please specify: ______________________________

Q20. Did this doctor refer you to a specialist / hospital for cancer treatment?

☐ 0 No

☐ 1 Yes

Q21. Did you see any other doctors for additional opinion for your cancer diagnosis?

☐ 0 No

☐ 1 Yes, **how many other doctors?** ____

Q22. Who was the first person you talked to about your cancer diagnosis?

☐ 1 I kept to myself

☐ 2 Partner / Husband / Wife

☐ 3 Other family members

☐ 4 Friends

☐ 99 Others, please specify: ________________________

SUBSECTION 4: CANCER JOURNEY (C: TREATMENT)

Q23. Who helped you make the final decision for your cancer treatment?

☐ 1 Myself

☐ 2 Partner / Husband / Wife

☐ 3 Other family members

☐ 4 Friends

☐ 99 Others, please specify: ____________________________

Q24. Who usually pays for your medical expenses for cancer?

Please TICK (✓) all the relevant boxes.

☐ 1 Self / Family members

☐ 2 Personal health insurance

☐ 3 Employer provided health insurance

☐ 4 Employer / Panel clinic (paid by employer)

☐ 5 Government / Pensioner

☐ 99 Others, please specify: ____________________________

Q25. Who normally accompanies you to see your healthcare provider during your cancer journey?

Please TICK (✓) all the relevant boxes.

☐ 1 I go on my own

☐ 2 Partner / husband / wife

☐ 3 Other family members

☐ 4 Friends

☐ 99 Others, please specify: ____________________________

Q26. Did you see any other doctors for additional opinion for your cancer treatment?

☐ 0 No

☐ 1 Yes, how many other doctors? ____

Q27. Have you ever missed any cancer treatment?

☐ 0 No

☐ 1 Yes

SUBSECTION 5: TRADITIONAL AND COMPLEMENTARY MEDICINE (T&CM)

Q28. Which of the following did you use after your cancer diagnosis was confirmed by a doctor?
Please TICK (✓) all the relevant boxes.

☐ 1 Malay herbs
☐ 2 Malay cupping
☐ 3 Malay massage
☐ 4 Acupuncture or Moxibustion
☐ 5 Chinese herbs
☐ 6 Tuina
☐ 7 Chinese cupping
☐ 8 Qi Gong
☐ 9 Ayurveda
☐ 10 Siddha
☐ 11 Unani
☐ 12 Yoga or Naturopathy
☐ 13 Homeopathy
☐ 14 Islamic Medical Practice
☐ 15 Mind – body Medicine Therapy (e.g. Hypnotherapy, Psychotherapy)
☐ 16 Biological based therapy (e.g. Aromatherapy, Nutritional Therapy)
☐ 17 Manipulative therapy (e.g. Chiropractic, osteopathy, reflexology, Thai massage, Swedish massage, Balinese/Javanese massage, shiatsu massage and aromatherapy massage)
☐ 18 Energy medicine (e.g. Reiki, aura metaphysic, colour vibration therapy)
☐ 99 Others, please specify: ____
☐ 0 Never tried any of the above. *(If you pick this, please ignore subsequent questions.)*

a. Who recommended the above options to you?
Please TICK (✓) all the relevant boxes.

☐ 1 No one
☐ 2 Family members
☐ 3 Friends
☐ 4 Medical staffs
☐ 5 Other cancer patients
☐ 99 Others, please specify: ____

SUBSECTION 5: TRADITIONAL AND COMPLEMENTARY MEDICINE (T&CM) (CONTINUED)

b. What made you use the above options?
Please TICK (✓) all the relevant boxes.

- ☐ 1 Could not afford conventional treatment. (e.g. surgery, radiotherapy, drugs / chemotherapy)
- ☐ 2 Attracted to the advertisement.
  (e.g. newspapers, magazine, flyers, social media, radio & TV announcement, etc)
- ☐ 3 Believe in its effectiveness to cure with less side effects.
- ☐ 4 Believe it can relieve my pain.
- ☐ 5 Believe it can treat the side effects of conventional treatment.
  (e.g. radiotherapy, drugs / chemotherapy)
- ☐ 6 Allow me to have more control over my illness than conventional medicine.
- ☐ 7 To make sure I receive all available treatment options.
- ☐ 8 It gives me positive hopes and peace of mind.
- ☐ 9 Unsure of its effect in treating cancer, so wanted to give it a try.
- ☐ 99 Others, please specify: ____

c. How much roughly have you spent in a month for traditional and complimentary medicine?

- ☐ 1 RM0 – RM99
- ☐ 2 RM100 – RM499
- ☐ 3 RM500 – RM1000
- ☐ 4 RM1001 – RM5000
- ☐ 5 RM5001 and above

d. Did you inform your doctors about the usage of the above option(s)?

- ☐ 1 Yes
- ☐ 0 No, **why?** *Please TICK (✓) all the relevant boxes.*
  - ☐ 1 The doctor will not understand why I am using it.
  - ☐ 2 The doctor did not ask me about it.
  - ☐ 3 I am unsure of the doctor’s reaction.
  - ☐ 4 I have not seen my doctor yet.
  - ☐ 5 I am afraid to tell the doctor.

SUBSECTION 5: TRADITIONAL AND COMPLEMENTARY MEDICINE (T&CM) (CONTINUED)
**e. Do you think the above options helped in treating your cancer?**<br>☐ 0 No<br>☐ 1 Yes

~~~THANK YOU FOR YOUR PARTICIPATION~~~

SECTION 2

SUBSECTION 1: DEMOGRAPHIC	
**Q1. Date of Birth**	☐☐ / ☐☐ / ☐☐☐☐<br>(DD / MM / YYYY)
**Q2. Postcode?**	☐☐☐☐☐
**Q3. Gender?**	☐ 1 Male<br>☐ 2 Female
**Q3a. Documented first (1st) symptom experienced date or duration**	☐ 1 DATE of 1st symptom experienced: ___ ___ / ___ ___ / ___ ___ ___ ___<br>**OR**<br>☐ 2 Duration : __________________________

SUBSECTION 2: DETAILS OF DIAGNOSIS	
BIOPSY RESULTS (PRE – SURGERY)	
**Q4.1. Date of Biopsy / Cytology Specimen taken**	☐☐ / ☐☐ / ☐☐☐☐<br>(DD / MM / YYYY)
**Q4.2. Date of Biopsy / Cytology Specimen Receipt by the Pathologist**	☐☐ / ☐☐ / ☐☐☐☐<br>(DD / MM / YYYY)
**Q4.3. Date of Biopsy / Cytology Pathology Report**	☐☐ / ☐☐ / ☐☐☐☐<br>(DD / MM / YYYY)
**Q4.4 Biopsy / Cytology Diagnosis**	
**Q4.5 Date of Patient informed of Biopsy / Cytology Diagnosis**	☐☐ / ☐☐ / ☐☐☐☐<br>(DD / MM / YYYY)

SUBSECTION 2: DETAILS OF DIAGNOSIS

SOLID ORGAN RESULTS (POST SURGERY)

**Q5.1. Date of Solid Organ Specimen taken**	☐☐ / ☐☐ / ☐☐☐☐<br>(DD / MM / YYYY)
**Q5.2. Date of Solid Organ Specimen Receipt by the Pathologist**	☐☐ / ☐☐ / ☐☐☐☐<br>(DD / MM / YYYY)
**Q5.3. Date of Solid Organ Pathology Report**	☐☐ / ☐☐ / ☐☐☐☐<br>(DD / MM / YYYY)
**Q5.4 Final Solid Organ Diagnosis**	
**Q5.5 Date of Patient informed of Solid Organ Diagnosis**	☐☐ / ☐☐ / ☐☐☐☐<br>(DD / MM / YYYY)

Q6. PATHOLOGICAL STAGING *(Fill all that is available)*

**CANCER STAGING**	☐ 1 Stage I<br>☐ 2 Stage II<br>☐ 3 Stage III<br>☐ 4 Stage IV

**TUMOUR (T)**		**FIGO Staging**<br>*(If Cervical)*	
**NODES (N)**		**Duke Staging**<br>*(If Colorectal)*	
**METASTASIS (M)**			

SUBSECTION 3: DETAILS OF TREATMENT	
**Q7. Date of First(1st) Treatment Received**	☐☐ / ☐☐ / ☐☐☐☐<br>(DD / MM / YYYY)

**Q8. Type of Treatments Option Given and Date of First(1st) Treatment Received**<br>*(*Please choose all relevant Treatments received <u>(Multiple Choice)</u>)*<br><br>*(*Please Enter the Following format for dates: <u>DD / MM / YYYY)</u>*	☐ 1 Surgery date: ___ ___ / ___ ___ / ___ ___ ___ ___<br>☐ 2 Radiotherapy date: ___ ___ / ___ ___ / ___ ___ ___ ___<br>☐ 3 Chemotherapy date: ___ ___ / ___ ___ / ___ ___ ___ ___<br>☐ 4 Hormonal Therapy date: ___ ___ / ___ ___ / ___ ___ ___ ___<br>☐ 5 Targeted Therapy date: ___ ___ / ___ ___ / ___ ___ ___ ___<br>☐ 6 Neo-Adjuvant Therapy date: ___ ___ / ___ ___ / ___ ___ ___ ___<br>☐ 7 Palliative Care date: ___ ___ / ___ ___ / ___ ___ ___ ___<br>☐ 8 Pregnancy EDD / actual birthdate: ___ ___ / ___ ___ / ___ ___ ___ ___<br>☐ 0 Refuse Treatment date: ___ ___ / ___ ___ / ___ ___ ___ ___
